# Redox and Hormonal Changes in the Transcriptome of Grape (*Vitis vinifera*) Berries during Natural Noble Rot Development

**DOI:** 10.3390/plants11070864

**Published:** 2022-03-24

**Authors:** Miklós Pogány, Tamás Dankó, Júlia Hegyi-Kaló, Evelin Kámán-Tóth, Dorottya Réka Szám, Kamirán Áron Hamow, Balázs Kalapos, Levente Kiss, József Fodor, Gábor Gullner, Kálmán Zoltán Váczy, Balázs Barna

**Affiliations:** 1 Centre for Agricultural Research, 2462 Martonvásár, Hungary; tamas.danko89@gmail.com (T.D.); toth.evelin@atk.hu (E.K.-T.); hamow.kamiran@atk.hu (K.Á.H.); kalapos.balazs@atk.hu (B.K.); or levente.kiss@usq.edu.au (L.K.); fodor.jozsef@atk.hu (J.F.); gullner.gabor@atk.hu (G.G.); barna.balazs@atk.hu (B.B.); 2 Food and Wine Research Institute, Eszterházy Károly Catholic University, 3300 Eger, Hungary; hegyi-kalo.julia@uni-eszterhazy.hu (J.H.-K.); vaczy.kalman@uni-eszterhazy.hu (K.Z.V.); 3 Georgikon Campus, Hungarian University of Agriculture and Life Sciences, 8360 Keszthely, Hungary; szam.dorottya.reka@gmail.com; 4 Centre for Crop Health, University of Southern Queensland, Toowoomba, QLD 4350, Australia

**Keywords:** grapevine, *Botrytis*, Furmint, noble rot, Tokaj, redox, hormone, abscisic acid

## Abstract

Noble rot is a favorable form of the interaction between grape (*Vitis* spp.) berries and the phytopathogenic fungus *Botrytis cinerea*. The transcriptome pattern of grapevine cells subject to natural noble rot development in the historic Hungarian Tokaj wine region has not been previously published. Furmint, a traditional white Tokaj variety suited to develop great quality noble rot was used in the experiments. Exploring a subset of the Furmint transcriptome redox and hormonal changes distinguishing between noble rot and bunch rot was revealed. Noble rot is defined by an early spike in abscisic acid (ABA) accumulation and a pronounced remodeling of ABA-related gene expression. Transcription of glutathione S-transferase isoforms is uniquely upregulated, whereas gene expression of some sectors of the antioxidative apparatus (e.g., catalases, carotenoid biosynthesis) is downregulated. These mRNA responses are lacking in berries exposed to bunch rot. Our results help to explain molecular details behind the fine and dynamic balance between noble rot and bunch rot development.

## 1. Introduction

*Botrytis cinerea* (gray mold), the filamentous plant pathogenic fungus, is responsible for losses in crop production of $10 billion to $100 billion worldwide [1]. Grapevine (*Vitis vinifera* L.) is the economically most important host of this pathogen. With the onset of ripening, grape berries exhibit a striking rise in their susceptibility to gray mold [2]. Cooler, rainy weather during harvest time often results in the development of disastrous bunch rot epidemics in vineyards unless the clusters are properly protected from *B. cinerea*. The interaction of *B. cinerea* with mature berries may also develop into a unique direction called noble rot, under the influence of special mesoclimatic conditions. This includes a short rainy period lasting for a few days followed by an extended period of warmer, windy, dry weather during daytime and cooler, misty, humid nights [3,4]. Berries of white grapevine cultivars affected by noble rot change their color to dark brown. They also shrivel and accumulate charming aroma components, whereas berries exposed to bunch rot develop a texture of soft decay with unappealing taste and smell [5,6]. Noble rotted grape berries are the main sources of flavor and odor in the outstanding botrytized dessert wines, such as the Trockenbeerenauslese of Germany, the Sauternes of France, or the Aszú of Hungary. Transcriptome studies of grape berry samples affected by natural or artificial noble rot induction have been published [7,8]. Here we present a subset of our RNAseq results using *Vitis vinifera* cv. Furmint samples exposed to natural noble rot development that were collected in a Tokaj vineyard in Hungary. Grapevine transcriptomic changes connected to the redox status and hormonal responses of botrytized grape berry cells are in the center of this work. Results of fungal gene expression will be presented in a later paper. Several reactive oxygen species (ROS) producing, scavenging, and signaling grapevine molecules have been connected to noble rot development [7,8]. The role of plant hormones, especially ethylene, abscisic acid, and auxins, has also been shown to be crucial in the processes of noble rot and berry ripening [7,8,9]. Redox-related transcriptional responses and transitions in plant hormone-associated gene activity are thoroughly investigated and discussed, focusing specifically on those factors that discriminate between noble rot and bunch rot.

Interactions between host plants and pathogens result in striking modifications in the cellular redox status and hormonal metabolism. Using our RNAseq datasets obtained from mature grape berries affected by noble rot and published gene expression data, we evaluated the mRNA expression patterns of various botrytized samples. The most intriguing question is whether the molecular mechanisms of gray mold or noble rot development could be partly unraveled by comparing hormone-associated and redox transcriptional patterns?

## 2. Results and Discussion

### 2.1. Transcriptional Pattern of Noble Rot in Furmint Correlates Well with Sémillon

**Figure 1:**
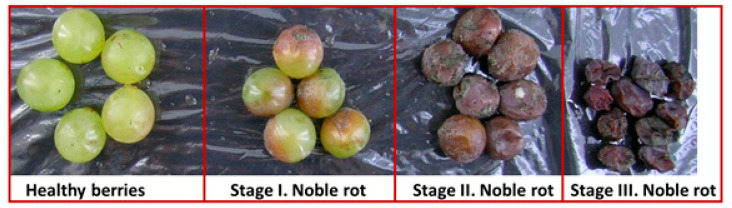
Healthy and botrytized *Vitis vinifera* cv. Furmint berries authentically represent samples used in this work. Besides healthy berries, three successive stages of noble rot were defined.

Four different types of *Vitis vinifera* cv. Furmint samples were collected starting with healthy berries followed by 3 consecutive stages of noble rot. Stage I was characterized by the partly botrytized epidermis and no shriveling; stage II berries showed fully colored epidermis and mild shriveling, while stage III exhibited all features of mature noble rot berries (fully shriveled with only limited presence of *B. cinerea* material on the surface) (Figure 1).

Dry weight (mg/g) of berries representing individual stages of noble rot were as follows: Healthy—0.228 ± 0.028 SD, Stage I. Noble rot—0.255 ± 0.008, Stage II Noble rot—0.285 ± 0.065, Stage III Noble rot—0.603 ± 0.065.

**Figure 2:**
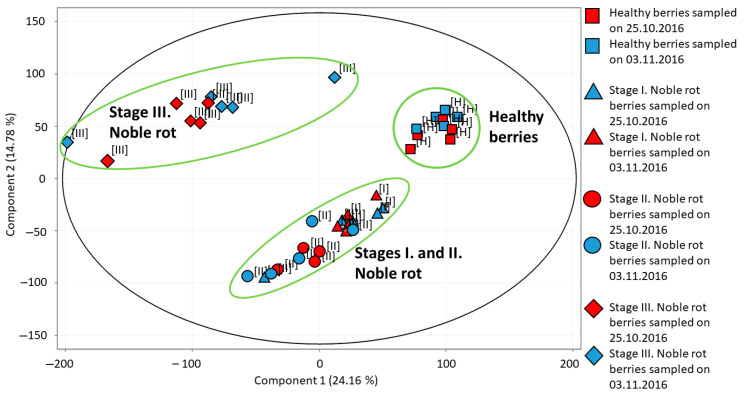
Principal component analysis of transcriptome profiles detected in various *Vitis vinifera* cv. Furmint berry samples. Healthy, stage I. noble rot, stage II. noble rot and stage III. noble rot samples were collected in October and November of 2016.

Using the transcriptome data of all grape berry samples, a principal component analysis (PCA) was performed (Figure 2). The first principal component (PC1) explaining 24.16% of the variance in our data separates healthy berries and stage III noble rot berries from the two earlier stages of noble rot (but it clusters the two earlier stages together). The second principal component (PC2) responsible for 14.78% of variance separates healthy and stage III noble rot samples from stages I and II samples, but it clusters healthy and stage III samples, and clusters stage I and stage II noble rot samples together. During the discussion of our transcriptome data, we are focusing on the transition between healthy berries and stage I–II noble rot berries. We could not distinguish clearly between stage I and stage II berries. Although the transcriptome pattern observed in stage III noble rot berries represents a unique group according to PCA, our prior berry vitality tests indicated a drastic decline in living berry exo- and mesocarp (skin and pulp) cells in Furmint stage III noble rot tissues. Therefore, transcriptome results of stage III noble rot samples were excluded from the discussion of results. PCA analysis did not separate samples collected at different sampling times (end of October or beginning of November) from each other. Therefore data of the two sampling dates were analyzed together in subsequent statistical processing of transcriptome results. 

To provide an overall view on the transcriptional reprogramming of Furmint grape berry cells under the influence of noble rot, we compared the pattern of activated and repressed genes to those reported in two earlier *Vitis vinifera*-*Botrytis* transcriptome studies [2,7]. In one study, Sémillon samples that were subjects of natural noble rot development were analyzed [7]. In another approach, Marselan grape samples collected in the vineyard were artificially inoculated with *B. cinerea* and incubated under bunch rot-inducing conditions [2]. Stage I. Furmint noble rot samples (Figure 1) were contrasted with stage II. Sémillon samples as they emerged most similar by macroscopic observation. Concerning the bunch rot experiment, mRNA expression results of mature Marselan berries collected 48 h after inoculation with *B. cinerea* were included.

**Figure 3:**
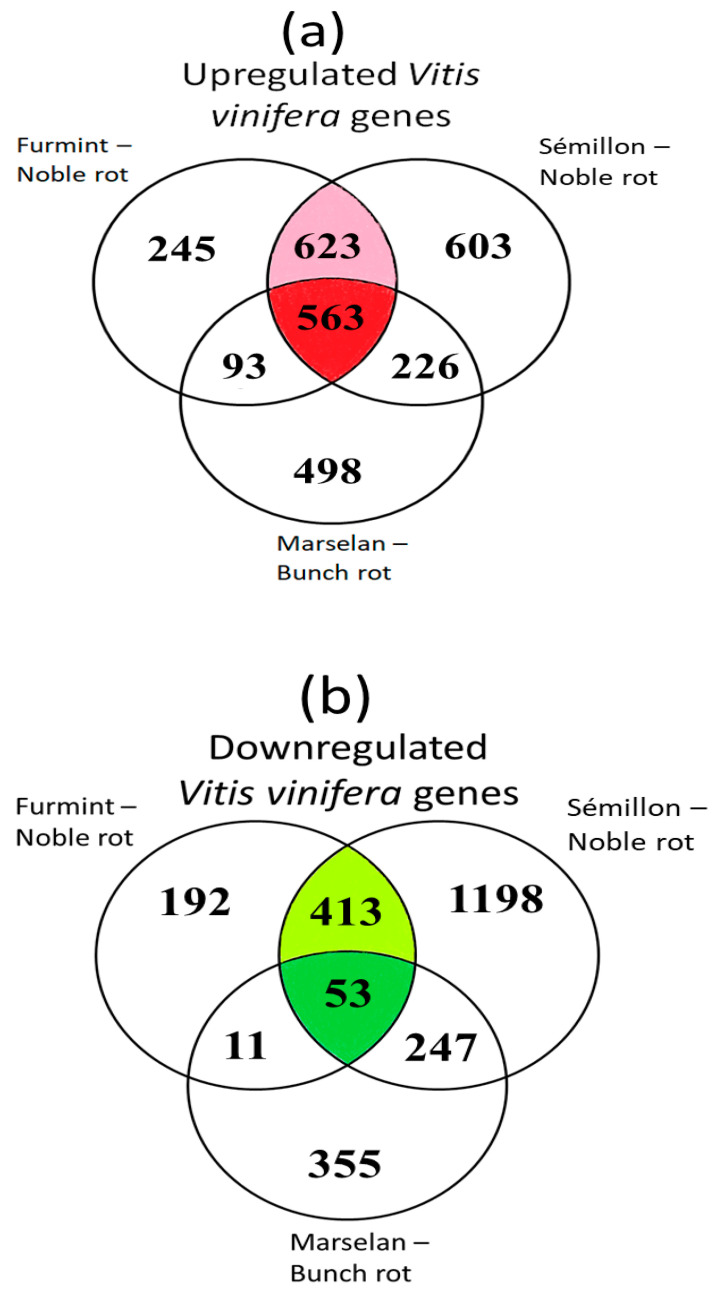
Venn diagrams show the overlap of differentially expressed genes (DEGs) during noble rot in Furmint and Sémillon and bunch rot in Marselan. Stage I. natural Furmint noble rot samples analyzed in this study were compared to stage II. natural Sémillon noble rot samples [7] and Marselan berries [2] that were collected 48 h after artificial inoculation with *B. cinerea*. (**a**) Upregulated DEGs in botrytized vs. healthy samples. (**b**) Downregulated DEGs.

Out of the 1524 upregulated grapevine transcripts in Furmint, 1186 (78%) were activated in Sémillon. A matching comparison between Marselan and Sémillon indicates only 57% overlap (Figure 3a). Examining the list of repressed transcripts, 70% of downregulated Furmint grapevine mRNAs showed decreases in Sémillon by noble rot. In contrast, Marselan berries affected by bunch rot exhibited a lower (45%) overlap with Sémillon noble rot samples regarding the list of transcripts with reduced abundance (Figure 3b).

These results suggest that Furmint and Sémillon samples (both exposed to noble rot-type botrytization) carry rather similar transcriptional patterns compared to bunch rot-affected Marselan samples.

Real-time RT-PCR results confirmed the reliability of our RNA-Seq analysis. The two assays provided comparable transcript abundance estimations with a correlation coefficient of 0.93 for Stage I. noble rot samples and 0.90 for Stage II. noble rot samples (Supplemental Table S3).

### 2.2. Abscisic Acid

**Figure 4:**
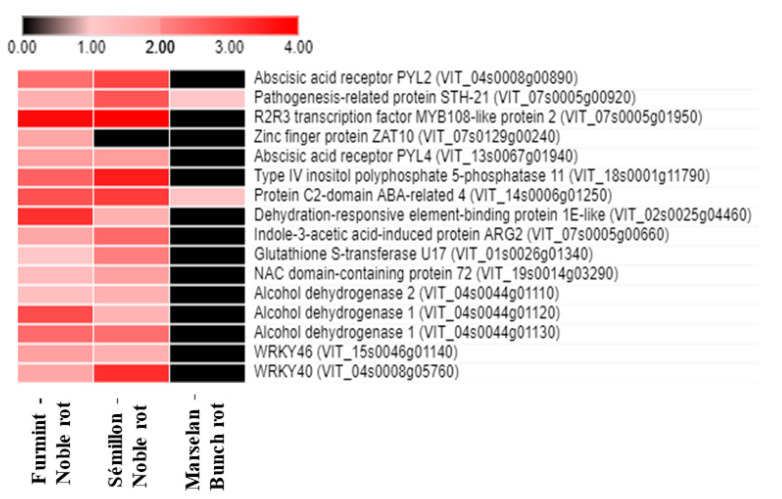
Heat map diagram showing the transcript abundance (Log2 fold change) of a set of ABA-associated *V. vinifera* genes in Furmint, Sémillon, and Marselan. Noble rot affects the expression of ABA-connected transcripts more robustly than bunch rot does. Transcriptome data for Furmint stage I. noble rot was analyzed in this study and Sémillon stage II. noble rot samples [7] are displayed as they appear most comparable by macroscopic observation of the berries. Concerning the bunch rot dataset, mRNA expression results of mature Marselan berries [2] collected 48 h after inoculation with *B. cinerea* are presented.

The plant stress and senescence hormone abscisic acid (ABA) is crucial in fruit ripening and *Botrytis*-induced berry responses [9,10]. Nevertheless, noble rot provoked a much more pronounced remodeling in the ABA-related transcriptional pattern compared to gray mold (Figure 4).

Seventy-five percent of ABA-associated responses detected in our noble-rotted Furmint samples were missing in grape berries affected by gray mold. These noble rot-specific transcriptional responses included the upregulation of transcripts encoding two ABA receptors, *PYL2* and *PYL4* (*VIT_04s0008g00890* and *VIT_13s0067g01940*). PYL2 and PYL4 are members of the PR10 group of pathogenesis-related proteins [11]. A PYL receptor-mediated crosstalk between ABA, JA, and anthocyanin accumulation was suggested [12,13].

Noble rot specifically activates mRNA expression of many ABA-responsive, drought tolerance-related transcription factors, such as the orthologs of *Arabidopsis Dehydration-Responsive Element-Binding Protein 1E* (*VIT_02s0025g04460*), *NAC72* (*VIT_19s0014g03290*), *AZF1* (*VIT_07s0129g00240*), and *Abscisic Acid-Insensitive 5* (*ABI5*) (*VIT_08s0007g03420*). Beyond the group of transcription factors, noble rot also specifically upregulates other osmotic stress-associated genes, including two *ABI5*-targeted *Late Embryogenesis Abundant 6* orthologs (*VIT_13s0067g01240* and *VIT_13s0067g01250*), an ortholog of *Arabidopsis Late Embryogenesis Abundant 3* (*VIT_07s0005g00660*) and *GSTU17*, a drought-stress responsive tau class *glutathione S-transferase* (*VIT_01s0026g01340*). Late Embryogenesis Abundant (LEA) proteins have been connected to cellular dehydration tolerance [14,15]. Another conspicuous ABA signaling response detected during noble rot is the repression of two *Hypersensitive to ABA1* (*HAB1*) orthologs (*VIT_04s0008g01420* and *VIT_11s0016g01780*). Gray mold development does not lead to the repression of grapevine *HAB1* genes in the berries [2,16]. HAB1 is a type 2C protein phosphatase and an important negative regulator of ABA signaling [17]. ABA receptors PYL2 and PYL4 were shown to establish a physical interaction with HAB1 [13,18].

**Figure 5:**
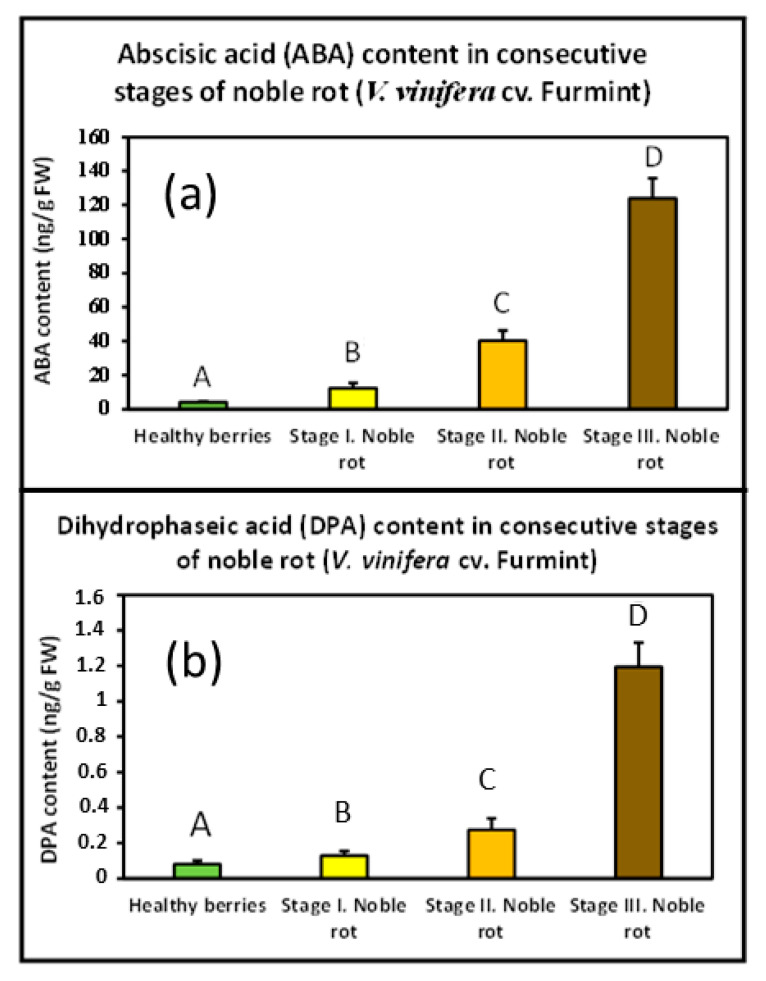
Noble rot-induced remodeling of abscisic acid (ABA) and dihydrophaseic acid (DPA) levels in Furmint samples. Values are means ± SEM, *n* = 6 for all groups. Data were log-transformed before analysis by ANOVA. Different letters indicate statistically significant differences at *p* ≤ 0.01. (**a**) ABA content in berries representing consecutive stages of noble rot; (**b**) DPA content in berries representing consecutive stages of noble rot.

Postharvest drying of Malvasia grapes results in elevated ABA content [19]. Whether noble rot was also accompanied by an increasing concentration of cellular ABA needed confirmation. Therefore, a UHPLC-MS/MS analysis was performed with our Furmint grape berry samples to monitor any transition in the ABA content during noble rot. Dihydrophaseic acid (DPA), a key metabolite of ABA catabolism, was also detected. However, the level of the other key metabolite phaseic acid (PA) did not reach the limits of detection in the examined samples. Noble rot triggered a sharp increase in ABA concentration from the beginning of the first visible symptoms (before any signs of shriveling), and its level kept exponentially growing in the berries till the end of the process. DPA content followed a similar pattern (Figure 5). Bunch rot (gray mold) disease of mature grape berries, however, did not seem to result in elevated ABA concentration [10]. Similar results were obtained with nearly mature kiwifruit after infection with *B. cinerea* [20].

Since noble rot is accompanied by a prompt and drastic surge of ABA formation and an obvious remodeling of ABA-connected gene expression, and these responses are lacking in berries affected by bunch rot, ABA seems to possess a central and distinctive role in noble rot development.

ABA seems to be a versatile player in the resistance of tomatoes against *Botrytis* [21]. On the one hand, it contributes to *Botrytis* susceptibility in tomato leaves [22]. But on the other hand, its positive role in *Botrytis* resistance was also suggested through the activity of Abscisic Acid-Induced MYB1 (AIM1) transcription factor [23].

### 2.3. Ethylene Response Transcription Factors

A vast majority of noble rot-affected grapevine genes associated with the gaseous plant hormone ethylene belong to the group of ethylene response transcription factors (ERFs). The expression of ERFs can be ethylene dependent or independent [24]. Noble rot apparently activated the transcription of grapevine *ERF109* (*VIT_03s0063g00460*), *ERF17* (*VIT_04s0008g02230*), *ERF98* (*VIT_05s0049g00500*), *ERF71* (*VIT_07s0005g00820*), *ERF11* (*VIT_07s0141g00690*), *ERF12* (*VIT_10s0003g00130*), *ERF16* (*VIT_11s0016g00670*), *ERF54* (*VIT_12s0059g00280*), *ERF3* (*VIT_12s0059g01460* and *VIT_19s0090g01080*), *ERF2* (*VIT_16s0013g00890*), *ERF10* (*VIT_18s0001g10150*) and *ERF23* (*VIT_18s0089g01030*). These ERFs were not induced in berries subjected to gray mold. ERF 109 is an APETALA2/ethylene response transcription factor that activates inhibitors of programmed cell death under salt stress and is involved in jasmonate-induced wound signaling in *Arabidopsis* [25,26]. ERF109 is also known as Redox Responsive Transcription Factor 1 (RRTF1). It is a component of a core redox signaling network in *Arabidopsis* [27]. ERF109 also activates transcription of a senescence-associated C2H2-type zinc finger transcription factor *ZAT12* in *Arabidopsis* [28]. RRTF1 and ZAT12 provide crosslinks between oxidative stress and abiotic/biotic stress responses in plants [29]. Grapevine orthologs of redox-responsive and interconnected *Arabidopsis* TF genes *ERF109* and *ZAT12* (*VIT_03s0063g00460* and *VIT_13s0019g00480*) were massively induced in berries subjected to noble rot. During bunch rot, however, *RRTF1* transcription remains unaffected, and *ZAT12* is only mildly upregulated. A citrus ortholog of *ERF17* has been reported to play a role in chlorophyll degradation and degreening of citrus fruits during ripening by establishing a physical interaction with the DRE motif of *pheophytin pheophorbide hydrolase* [30]. Arabidopsis ERF71 is a major regulator of hypoxia response [31,32]. In grape berries, not only the *Vitis vinifera* ortholog of ERF71 was activated during noble rot, but also its predicted interacting partners, such as *grapevine cysteine oxidase 2* (*VIT_08s0056g01300*, *VIT_14s0108g00090*, *VIT_17s0000g05790*) acting as oxygen sensors [33], the *non-symbiotic hemoglobin 1* (*VIT_03s0063g01960*) acting as a scavenger of nitric oxide [34] or the later discussed *alcohol dehydrogenase 1*. These interacting factors of ERF 71 have been conclusively associated with hypoxia stress adaptation in plants [35,36,37].

### 2.4. Auxin

The expression of several genes related to auxin metabolism or signaling was significantly affected by noble rot. Two *auxin-responsive protein*-encoding transcripts (*VIT_08s0058g01160, VIT_09s0002g00650*), an *ARG7-like* and a *SAUR50* gene were massively upregulated. Furthermore, an *IAA-amino acid hydrolase ILR1-like 6* gene (*VIT_18s0001g02570*) and a *type IV inositol polyphosphate 5-phosphatase 11* gene (*VIT_18s0001g11790*) were also induced during noble rot. Nevertheless, transcriptional changes that were unique to noble rot mostly included downregulated mRNA responses, such as genes encoding an *auxin-induced ubiquitin superfamily protein 22D* (*VIT_14s0030g02310*), an *auxin transporter-like protein 2* (*VIT_03s0038g02140*), or an *auxin response factor 18* (*VIT_13s0019g04380*).

This is in accord with Lovato et al. [8], where RNA-Seq and subsequent Mapman analysis revealed the suppression of the auxin pathway in Garganega grape berries exposed to artificial noble rot.

### 2.5. Cytokinin

Noble rot affected the expression of only a handful of cytokinin-related transcripts. The most notable noble rot-specific mRNA response (that does not appear during bunch rot) is the repression of the cytokinin receptor transcript *histidine kinase 4* (*VIT_01s0011g06190*).

### 2.6. Salicylic Acid

Two grapevine *salicylic acid-binding protein 2* isoforms (*VIT_00s0253g00140*, *VIT_00s0253g00150*) were upregulated in Furmint in a noble rot-specific manner. The *Arabidopsis* ortholog of *VIT_00s0253g00140* (*AtMES1*) possessed methyl salicylate esterase activity with some additional preference for methyl jasmonate as a substrate as well. Expression of AtMES1 complemented systemic acquired resistance deficiency in salicylic acid-binding protein 2-silenced tobacco [38]. In addition, two *salicylate 1-O-methyltransferase* isoforms (*VIT_12s0057g01060*, *VIT_12s0057g01070*) were repressed in Furmint berries during noble rot.

### 2.7. Jasmonic Acid

Concerning transcripts that regulate jasmonic acid metabolism, two *jasmonate O-methyltransferase* isoforms (*VIT_18s0001g12890*, *VIT_18s0001g12900*) were markedly activated as a result of noble rot development in Furmint. This response is not specific to noble rot because Marselan berries subjects of bunch rot also showed strong induction of these two transcripts [2]. Jasmonate O-methyltransferases catalyze the methylation of jasmonate into methyl jasmonate, a plant volatile that serves as an important cellular regulator conveying diverse developmental processes and defense responses. Interestingly, a *hydroxyjasmonate sulfotransferase* gene (*VIT_13s0084g00240*) is highly suppressed in Furmint in a noble rot-specific manner. Sulfonation would inactivate jasmonic acid in plants, and inhibition of this reaction maintains the level of functional jasmonic acid in the berries during noble rot [39].

### 2.8. Analysis of Genes Modulating the Redox State during Noble Rot

A subset of our transcriptomic data associated with cellular redox status and regulation was also comprehensively evaluated because previous extensive gene expression studies have already identified some important aspects of redox-related mRNA responses during noble rot [7,8].

#### 2.8.1. Glutathione S-Transferases

**Figure 6:**
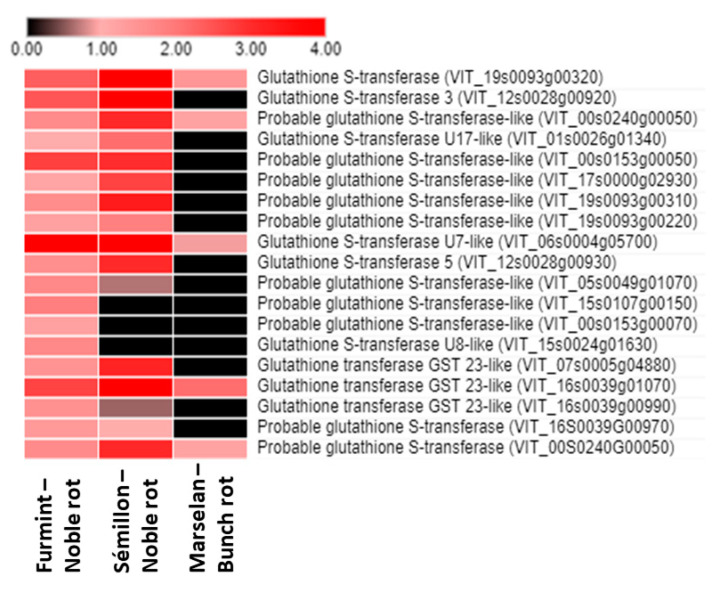
Heat map diagram showing the transcript abundance (Log2 fold change) of a set of *V. vinifera glutathione S-transferase* (*GST*) genes in Furmint, Sémillon, and Marselan cultivars. Fold induction results are presented after Log2 transformation. Noble rot affects the expression of *GST* transcripts more robustly than bunch rot does. Transcriptome data for Furmint stage I. noble rot was analyzed in this study and Sémillon stage II. noble rot samples [7] are displayed as they appear most comparable by macroscopic observation of the berries. Concerning the bunch rot dataset, mRNA expression results of mature Marselan berries [2] collected 48 h after inoculation with *B. cinerea* are presented.

One of the most pronounced redox-related (and global) noble rot-specific transition in the grape berry transcriptome is the uniform upregulation of the gene expression of ***glutathione S-transferase*** (*GST*) isoforms. In our experiments, several *GST* genes were markedly induced in grape berries during the progress of noble rot infection. The most significant induction was observed in the case of the *VIT_06s0004g05700* gene, which encodes a tau class GST. Furthermore, three tau class *GST* genes (*VIT_00s0153g00050*, *VIT_16s0039g01070*, *VIT_19s0093g00320*) and a phi class *GST* (*VIT_12s0028g00920*) were also markedly induced in the noble-rotted berries. Taken together, noble rot development in Furmint berries led to the marked induction of 19 *GST* isoforms (Figure 6), and in sharp contrast, gray mold infection resulted in a slight activation of only 5 *GST* genes [2]. The ‘classical’ function of GSTs is the cellular detoxification of a wide variety of endobiotic and xenobiotic substrates by conjugating them to glutathione. Generally, tau class *GST* genes (*GSTU*s) can be induced by different abiotic stress factors [40] as well as by microbial infections [41], and they have an important role in the protection of plants against oxidative damage [42]. A tau class GST (GSTU10) was shown to participate in the transport of trans-resveratrol out of grapevine cells [43]. The expression of the several phi class grapevine *GSTs* (*VvGST2*, *VvGST3*, and *VvGST4*) was markedly higher in post-veraison berry skins concomitantly with the accumulation of anthocyanins [44]. These GSTs showed a non-enzymatic carrier (ligandin) function, by which they participate in the transport of anthocyanins and proanthocyanidins (tannins) from the cytosol to the plant vacuole [44,45]. Members of the lambda class GSTs (GSTLs) catalyze deglutathionylation reactions via a catalytic cysteine residue [46].

#### 2.8.2. Crocetin Glucosyltransferases

Five crocetin glucosyltransferase genes were robustly activated during the development of *B. cinerea* infection. The highest induction was observed in the case of *VIT_05s0062g00310* (183-fold induction), while the genes *VIT_05s0062g00300*, *VIT_05s0062g00270*, *VIT_05s0062g00710*, and *VIT_05s0062g00700* were also markedly induced. Crocetin and its glycosylated derivatives, crocins are antioxidative apocarotenoids, which also provide a red color to saffron [47,48]. In addition, crocetin exhibits various health-promoting properties including anti-tumor, neuroprotective, anti-diabetic, anti-inflammatory and anti-hyperlipidemia effects [49,50]. The final step in the biosynthesis of the 20-carbon esterified carotenoid crocin is the transformation of the insoluble crocetin into a soluble and stable storage form by glucosylation [47]. In grapevine berries, the exact biochemical roles of crocetin and crocin are largely unknown.

#### 2.8.3. Changes in Transcript Levels of Phenylpropanoid Pathway Components

Induction of the phenylpropanoid pathway, including the accumulation of flavonoids is a typical metabolic response of plant cells exposed to pathogen infection [51]. Phenylpropanoid molecules, such as the non-flavonoid stilbenes, lignans, and phenolic acids or the flavonoid flavones, flavonols, flavanones, flavanols, anthocyanins, and chalcones are significant constituents of the non-enzymatic antioxidant pool of plant cells [52,53].

**Figure 7:**
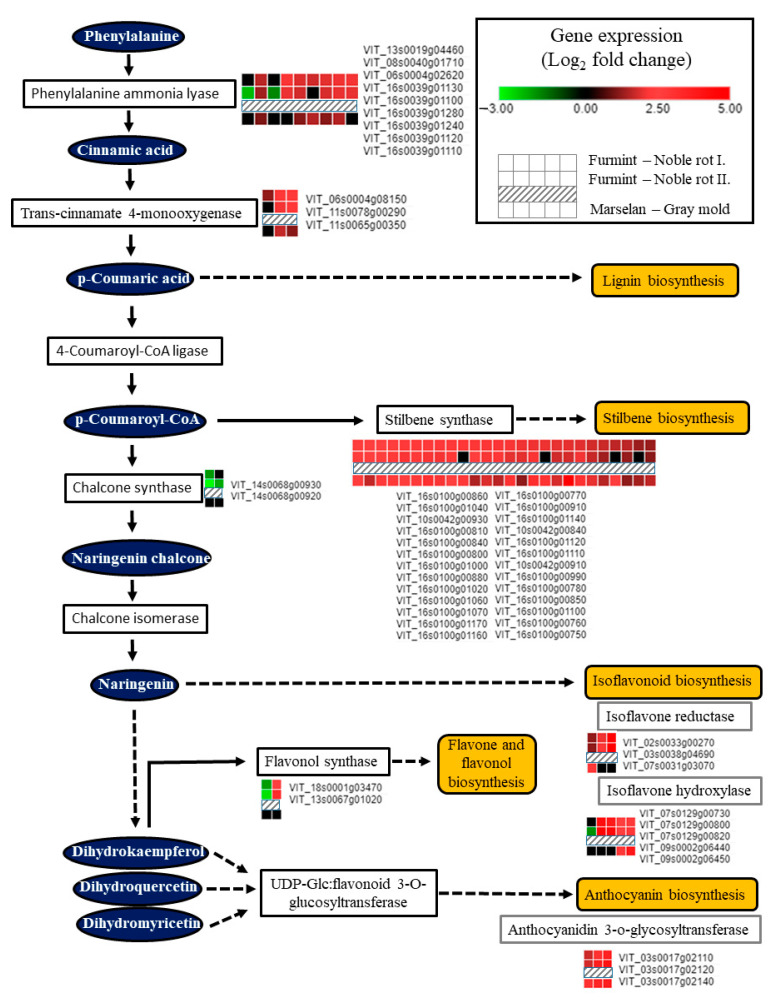
Transcription of genes encoding components of the phenylpropanoid pathway is greatly affected by noble rot in Furmint (analyzed in this study). Bunch rot also results in a marked transcriptional change observed in Marselan berries [2].

Noble rot development triggered the transcription of enzymes responsible for the initial steps of the phenylpropanoid pathway (Figure 7). Several isoforms of *phenylalanine ammonia-lyase* and *trans-cinnamate 4-monooxygenase* were activated, contributing to the formation of key phenolic compounds, cinnamic acid, and p-coumaric acid. Induction of these two enzymes at the mRNA level is not specific to noble rot but rather a common transcriptional response of grape berry cells elicited by *B. cinerea* infection [2,16]. *Stilbene synthase* (*STS*) isoforms encoded in two massive clusters on chromosomes 10 and 16 have been uniformly upregulated in our Furmint noble rot samples similar to berries subjected to typical bunch rot disease. Two *chalcone synthase* (*CHS*) isoforms (*VIT_14s0068g00920* and *VIT_14s0068g00930*), on the other hand, were inversely regulated between noble rot (repression) and bunch rot (activation). STS and CHS catalyzed branches of the phenylpropanoid pathway might be competing with each other in berries during noble rot, as it was reported before in downy mildew-infected grapevine leaves [54]. Two ***chalcone synthase*** (*CHS*) genes (*VIT_14s0068g00920* and *VIT_14s0068g00930*) are specifically downregulated during noble rot (Figure 7). In bunch rot, these two *CHS* isoforms are activated [16]. It is conceivable that they control branches of the flavonoid biosynthesis pathway such as Flavonol synthase 1 (FLS1) and flavonol synthesis that are responsible for the reduced flavonol synthase activity characteristic of noble-rotted berries [7].

Interestingly, *chalcone synthase* (*CHS*) and *stilbene synthase* (*STS*) transcripts are differentially regulated during noble rot but are uniformly activated in bunch rot, providing another example of competition between CHS and STS pathways in grapevine. Similar competition between these two pathways has been found during development, abiotic stress, or downy mildew infection, which may direct the flow of carbon toward stilbene biosynthesis at the expense of flavonol formation [54].

*VIT_18s0001g03470*, the *V. vinifera* ortholog of *Arabidopsis Flavonol synthase 1* (*FLS1*), was repressed in berries subjected to noble rot (Figure 7). *FLS1* is the most important flavonol synthase isoform in *Arabidopsis* with a defining role in flavonol levels compared to other isoforms [55]. Similarly, decreased flavonol synthase activity was detected in Sémillon berries during noble rot development [7]. Bunch rot, however, did not suppress the transcription of *vvFLS1* (*VIT_18s0001g03470*) in Trincadeira and Marselan berries [2,16]. Interestingly, Arabidopsis orthologs (*JAO1* and *JAO2*) of two noble rot-induced *flavonol synthase* transcripts (*VIT_08s0105g00380* and *VIT_13s0067g01020*) have been recently shown to inactivate jasmonic acid and down-regulate immunity upon *B. cinerea* infection [56,57]. Noble rot development in the white grape cultivar Sémillon was associated with an unusual accumulation of anthocyanins [7]. In accordance, a cluster of *anthocyanidin 3-O-glucosyltransferase* isoforms encoded on chromosome 3 (*VIT_03s0017g02110*, *VIT_03s0017g02120*, *VIT_03s0017g02140*) exhibited substantial upregulation during noble rot in white-skinned Furmint berries. Increased anthocyanin formation is a typical hallmark of ripening red-skinned cultivars, a phenomenon that is normally missing in berries of white-skinned grapevine cultivars [7]. Lastly, transcripts involved in isoflavonoid metabolisms, such as isoflavone reductase and isoflavone hydroxylases, were consistently upregulated due to noble rot (Figure 7). These enzymes have been linked with the synthesis of pterocarpan phytoalexins in legumes [58].

#### 2.8.4. Further Components of the Grape Berry Antioxidative System

**Figure 8:**
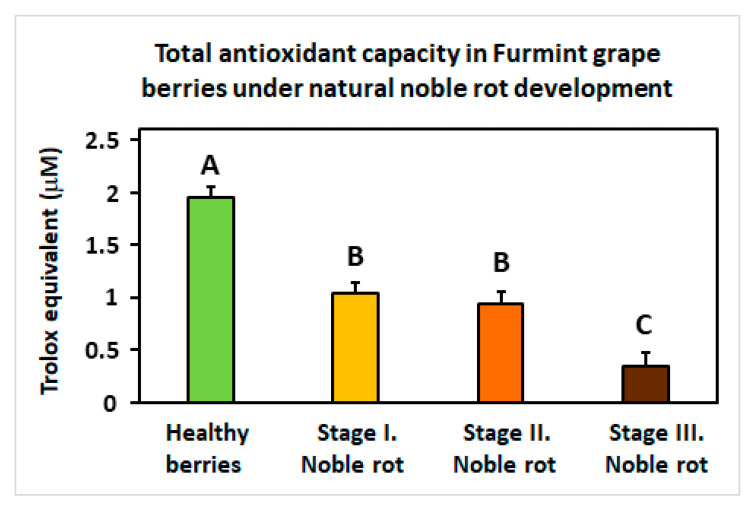
The total antioxidative capacity of grapevine berries at four different stages (Healthy, Stages I.–III. of Noble rot) of botrytization. The antioxidative capacity is expressed in Trolox equivalents. Values are means ± SEM, *n* = 5 for all groups. Different letters indicate statistically significant differences at *p* ≤ 0.01.

Another remarkable redox-associated response of noble rot is the specific inhibition of some sectors of the plant antioxidative system. Peroxisome-located ***catalase*** isoforms (*VIT_00s0698g00010* and *VIT_04s0044g00020*) are repressed, and concomitantly, a set of chloroplastic ***carotenoid biosynthesis* genes** (e.g., *VIT_05s0062g01110*; [59]) is uniformly down-regulated. These antioxidant responses are consistently lacking in berries during bunch rot. In the meantime, noble rot development triggers the gradual decline of **total** (Trolox equivalent) **antioxidant capacity** in Furmint berries (Figure 8). Components of the ascorbate-glutathione cycle do not show apparent transcriptional changes. Exploring the most reasonable causes that may explain this observed decrease in antioxidant functions compared to bunch rot, the conclusively proved accumulation of anthocyanin phenylpropanoids in noble-rotted berries may be responsible for the downregulation of other antioxidant molecules. Anthocyanins serve as optical filters that protect chloroplasts from photoinhibition light fluxes, such as in leaves of deciduous trees that turn red in the autumn [60,61,62]. In addition, anthocyanins are also free radical scavengers themselves that may replace other antioxidant systems in berries during noble rot, resulting in a decrease in (Trolox equivalent) net antioxidant capacity [63]. It should be noted, however, that bunch rot also induces a color change and some increase in the anthocyanin content of berries [16].

### 2.9. Transcription of Redox Signaling-Connected and Redox-Regulated Genes in Noble Rot

#### 2.9.1. NPR1 Interactors NIMIN-1 and NIMIN-2

Non-expression of PR genes 1 (NPR1 or NIM1) is a master redox sensor in plant defense [64]. Although transcription of *V. vinifera NPR1/NIM1* isoforms did not seem to be activated in Furmint berries during noble rot, *V. vinifera* orthologs of NPR1-interacting ***NIMIN-1*** and ***NIMIN-2*** are among the most highly upregulated genes in our samples. Moreover, this robust transcriptional response is noble rot specific because in bunch rot samples *NIMIN* gene activation was lacking [2,16].

NPR1 protein plays a crucial role in defense responses against phytopathogens by integrating redox and salicylic acid signaling [64]. Transcription of two negative regulators of NPR1, ***NIMIN-1*,** and ***NIMIN-2*** (*VIT_07s0005g02070*, *VIT_01s0011g03430*), is drastically upregulated during noble rot but not in bunch rot. Since NIMIN proteins are suppressors of some salicylic acid-induced responses and systemic acquired resistance [65,66] and salicylic acid signaling typically represses jasmonic acid-mediated signaling pathways [67], activation of NIMIN-1 and NIMIN-2 may therefore contribute to *Botrytis* resistance during noble rot in comparison with bunch rot. Moreover, *TGA9* (*TGACG MOTIF-BINDING PROTEIN 9*), a redox-sensitive bZIP transcription factor (*VIT_06s0080g00360*), and its nuclear interactor *ROXY1*, a CC-type glutaredoxin (*VIT_01s0146g00220*), are also transcriptionally activated in a noble rot-specific manner. TGA9 is connected to ROS-mediated Pattern-Triggered Immunity [68], and ROXY1 contributes to hydrogen peroxide accumulation and *B. cinerea* susceptibility in *Arabidopsis* [69]. In addition to NIMIN proteins, redox regulator TGA transcription factors also modulate NPR1 activity [70].

#### 2.9.2. MCP1 Metacaspase

Lesion Simulating Disease 1 (LSD1) is an important regulator of ROS and hormonal homeostasis in plants [71].

**Metacaspase 1** (**MCP1**) is a crucial component of programmed cell death development in plant cells. *Arabidopsis* MCP1 physically interacts with LSD1 and contributes to superoxide-initiated cell death in juvenile plant tissues, but in aging cells, it counteracts the process of cell death by regulating autophagy and vacuolar lysis [72,73]. Grapevine *MCP1* isoforms (encoded by *VIT_16s0013g00210* and *VIT_16s0013g00220*) are transcriptionally activated during noble rot of the berries. Bunch rot, on the other hand, does not lead to the induction of *MCP1* [2,16]. In ripe berries, the second function of MCP1 is more likely to be utilized by the plant cells, and in senescent tissues, MCP1 suppresses *B. cinerea*-induced tissue necrosis [73]. VvMCP1, a redox-associated plant metacaspase might therefore adjust the spread of programmed cell death in noble rot and orchestrate symptom development caused by the fungus.

#### 2.9.3. Transcripts of Redox-Associated Chloroplastic Proteins Are Suppressed

A uniform down-regulation of transcripts encoding redox-associated chloroplastic proteins has been observed in berries affected by noble rot. With a few exceptions, these genes listed in Table 1 correspond with transcripts of redox-sensitive starch metabolizing enzymes and redox-regulated proteins localized in the chloroplast of the model plant *Arabidopsis thaliana* [74,75]. Decreased mRNA abundance of chloroplastic redox proteins is a characteristic of noble rot, and it is not typical of berries exposed to bunch rot.

#### 2.9.4. Alcohol Dehydrogenase 1

Grapevine orthologs (*VIT_04s0044g01110*, *VIT_04s0044g01120*, *VIT_04s0044g01130*) of *Arabidopsis Alcohol Dehydrogenase 1* (*AtADH1*) are distinctively upregulated during noble rot, which response does not occur in berries under bunch rot development. Alcohol dehydrogenases catalyze the last step of the ethanol fermentation pathway (the reversible conversion of acetaldehyde to ethanol) used by plants to cope with energy deficiency during hypoxic stress, and the activity of AtADH1 is controlled by redox cues [82,83].

AtADH1 is not only redox-regulated, but it also contributes to the ABA sensitivity and abiotic (drought, salinity) as well as biotic (*Pseudomonas syringae* pv. *tabaci* DC3000) stress resistance of plants. In addition, *AtADH1* overexpression results in the accumulation of soluble sugars in leaves [84]. Grapevine orthologs of *AtADH1* interactors appearing in the STRING v11 database, such as *Pyruvate Decarboxylase-2* (*VIT_08s0217g00100*) and two *aldehyde dehydrogenases* (*VIT_06s0004g02060*, *VIT_14s0066g01550*), are also differentially expressed in a noble rot specific manner [85]. *VvADH1* isoforms might be partly responsible for sugar accumulation characteristic of berries enduring noble rot [86], and they might be regulated by redox signals and ABA.

#### 2.9.5. WRKY Transcription Factors

Transcript levels of two WRKY transcription factors, *WRKY40* and *WRK46*, were distinctively elevated during noble rot. *VIT_15s0046g01140*, a grapevine ortholog of *AtWRKY46* exhibited increasing abundance throughout the development of noble rot compared to bunch rot. Similarly, *VIT_04s0008g05760*, an *AtWRKY40* ortholog, was upregulated in Furmint berry samples subjected to noble rot. Both *AtWRKY46* and *AtWRKY40* are redox-responsive [29,87]. In *Arabidopsis, thaliana* WRKY40 interacts with the aforementioned TF ERF109/RRTF1 by binding to the W box sequence of its promoter region [28,88]. ROS-generating stimuli from necrotrophic infections stimulate *ERF109* expression, while biotrophic and mutualistic microbes repress *ERF109* expression [28]. WRKY46 and WRKY40 are also important regulators of ABA signaling and osmotic stress responses [89,90,91]. It should be noted that some other *WRKY TF* genes, such as *WRKY7* (*VIT_07s0031g00080*, *VIT_18s0001g10030*), *WRKY9* (*VIT_12s0055g00340*), *WRKY22* (*VIT_15s0046g02190*), and *WRKY70* (*VIT_08s0058g01390*, *VIT_13s0067g03140*) with a less obvious connection to redox regulation or ABA signaling have also displayed noble rot-specific activation in our Furmint samples.

## 3. Materials and Methods

### 3.1. Plant Material and Sampling

Healthy and botrytized *Vitis vinifera* cv. Furmint berry samples were collected in Mád (Tokaj wine region), Hungary in vineyard Betsek, 48°11′16″ N 21°19′03″ E, on 25 October and 3 November 2016. Collection and handling of the berries were the same at both sampling times. Four different samples were collected: healthy berries, stage I noble rot, stage II noble rot and stage III noble rot berries (Figure 1). Five independent biological replicates were collected at both time points in 50 mL centrifuge tubes for all four types of samples, each tube containing 5 (healthy), or 10 (stage I and II) or 15 (stage III) berries. Each stage of noble rot was represented by a total of ten biological replicates. Samples were frozen immediately in liquid nitrogen, transported in dry ice, and stored at −70 °C in an ultra-low temperature laboratory freezer.

### 3.2. RNA Extraction

Berries were ground to a fine powder in liquid nitrogen by a Retsch laboratory ball mill. Considering the uneven dry weight of the berry samples, varying amounts of frozen plant powder were weighed into new 50 mL centrifuge tubes, 800 mg for healthy and stage I berries, 500 mg for stage II berries and 250 mg for stage III berries. Total RNA extraction was carried out according to Reid et al. [92]. Twenty milliliter extraction buffer [300 mM Tris HCl (pH 8.0), 25 mM EDTA, 2 M NaCl, 2% CTAB, 2% PVPP, 0.05% spermidine trihydrochloride, 2% β-mercaptoethanol (administered immediately before use)] was added to powdered samples which were incubated at 65 °C for 10 min and vortexed every 2 min. After that, mixtures were extracted twice with equal volumes of chloroform: isoamyl alcohol (24:1), thoroughly vortexed, and centrifuged at 4800× *g* for 10 min at 4 °C. The supernatant (10 mL) was transferred to a new 50 mL centrifuge tube and combined with 0.1 vol 3M NaOAc (pH 5.2) and 0.6 vol ice-cold isopropanol, mixed and kept for 30 min at −70 °C. The nucleic acid pellets were recovered by centrifugation at 4800× *g* for 30 min at 4 °C. The supernatant was removed, and the remaining pellet was washed with 5 mL of 70% EtOH, then centrifuged at 4800× *g* for 15 min at 4 °C. The liquid was discarded and the precipitate was dried with a lyophilizer.

The nucleic acid pellets were dissolved in 1 mL TE (Tris-EDTA) buffer (pH 7.0) and transferred to new 2 mL microcentrifuge tubes. Selective RNA precipitation was achieved by adding 0.3 vol of 8M LiCl and a subsequent overnight incubation at 4 °C. Total grapevine and fungal RNA were harvested by centrifugation at 21,000× *g* for 30 min at 4 °C followed by a washing step with 500 μL 70% ice-cold EtOH and finally drying by a lyophilizer. Isolated RNA pellets were dissolved in 42 μL nuclease-free ultrapure water, and RNA quality was first evaluated using a NanoDrop 1000 spectrophotometer (Thermo Fisher Scientific, Waltham, MA, USA). Then all samples were subjected to DNase treatment using the DNA-free™ DNA Removal Kit (Invitrogen, Thermo Fisher Scientific, Waltham, MA, USA). Total RNA sample quality was analyzed more precisely on an Agilent BioAnalyzer) using Eukaryotic Total RNA Nano and Pico Kit (Agilent Technologies, Palo Alto, CA, USA) according to the manufacturer’s protocol. Samples with RNA integrity number (RIN) value > 7 were accepted for the library preparation process.

### 3.3. RNA-Seq Library Preparation and Sequencing

RNA-Seq libraries were prepared from total RNA using a TruSeq RNA Sample preparation kit (Illumina, San Diego, CA, USA) according to the manufacturer’s instructions. Shortly, poly-A RNAs were captured by oligo-dT conjugated magnetic beads then the eluted mRNAs were fragmented at 94 °C. First-strand cDNA was synthesized by random priming reverse transcription, and after the second strand synthesis step, double-stranded cDNA was produced. Following repairing ends, A-tailing and adapter ligation steps, adapter-ligated fragments were amplified in enrichment polymerase chain reaction (PCR), and lastly, a double-stranded mRNA sequencing library was generated. Sequencing runs were performed on Illumina NextSeq500 instrument using single read, 75bp-long sequencing mode and generating 18–20 million sequencing reads for each sample.

### 3.4. Primary and Secondary Bioinformatics Analyses

The quality of fastq files was evaluated by using FastQC v 0.11.7 software. After that, raw sequence reads were aligned to the reference genome (*Vitis vinifera* IGGP_12x genome version downloaded from the Ensembl Plants). STAR v2.5.4b bioinformatics tool was used for the mapping, and bam files were created. StrandNGS software (Agilent Technologies) was used for selecting lists of transcripts, which show differential mRNA abundance between conditions. Bam files of all individual biological samples were transferred, and raw expression data were normalized using the DESeq2 tool. To identify differentially expressed genes between healthy grape berry samples and various stages of noble rot, an ANOVA test with subsequent Tukey *post hoc* test and Benjamini-Hochberg FDR for multiple testing correction was used. Gene expression records that passed the following criterion were used: *p*  ≤  0.05 and −1  ≥  Log2 fold change (FC)  ≥  1. Supplemental Table S1 contains all the grapevine gene loci discussed in our work, their exact transcript abundance fold change values between healthy grape berry samples and individual noble rot stages, as well as the ANOVA and Tukey *p* values for each transcript or pairwise comparison. RNA-seq raw data were deposited at ArrayExpress (E-MTAB-11205) and the European Nucleotide Archive (PRJEB48949).

### 3.5. Validation of RNA-Seq Results

RNA-Seq results were verified by real-time RT-PCR analysis of the expression of 5 grapevines and 5 *B. cinerea* genes as described before [93]. First-strand cDNA was synthesized with the First Strand cDNA Synthesis Kit (Thermo Fisher Scientific) and used as a template for real-time PCR analysis in five-fold dilution. Relative quantification analysis was carried out using the comparative 2^−ΔΔCt^ method [94]. To assess the level of gene expression, results were normalized using Ct values from the cDNA amplification of the constitutively expressed *V. vinifera* actin gene *VIT_08s0007g06520* [92] and *B. cinerea* β-tubulin gene *BCIN_01g08040* [95]. Results of RNA-Seq validation, primer sequences and Real-time RT-PCR parameters are shown in Supplemental Tables S2 and S3.

### 3.6. Functional Annotation

Ensembl identifiers and transcript sequences of differentially expressed *Vitis vinifera* genes were used to collect functional information in KEGG, PFAM, GO, and TAIR databases as recently described [96]. Protein-protein interactions were predicted by the STRING v11 database.

### 3.7. UPLC-MS/MS Analysis of Abscisic Acid and Related Metabolites

Extraction and chromatographic analyses of abscisic acid (ABA) and its related metabolites, namely phaseic acid (PA) and dihydrophaseic acid (DPA), were carried out according to Vrhovsek et al. [97] and Pál et al. [98] with slight modifications.

Grape samples were homogenized in liquid N_2_, stored at −80 °C until preparation, 0.2 g (F.W.) were weighted into 2 mL safety Eppendorf tubes containing two borosilicate inert glass beads (df = 3 mm). 100 µL water was added to stage III. noble rot samples to compensate for their low water content. Samples were spiked with 20 ng [^2^H_6_](+)-*cis,trans*-abscisic acid (OlChemIm s.r.o. Olomouc, Czech Republic) before extraction to serve as an internal standard. Extraction was carried out with 2 × 1.5 mL of methanol: water (2:1 *v*/*v*%) by vortex mixing for 10 s, followed by shaking for 3 min with 1250 rpm in a cryo-cooled rack using a miniG1600 (SPEX SamplePrep.; Metuchen, NJ, USA). After centrifugation (16,500× *g*; 4 °C; 10 min), supernatants were collected and pooled. To remove lipids and carotenoids, 1.2 mL of the supernatant was partitioned by adding 0.6 mL of n-hexane and vortexed for 2 × 10 s. Phases were separated by centrifugation (10,000× *g*; 4°; 10 min) and the bottom phase of methanol: water was collected and filtered through 0.22 µm PTFE syringe filters before analysis.

After injecting 2 µL separation was achieved on a Waters (Milford, MA, USA) Acquity I-class UPLC equipped with an HSS T3 column (1.8 μm; 100 mm × 2.1 mm; Waters) at 40 °C in a 15 min water: acetonitrile (0.1 *v*/*v*% FA) gradient. For detection, a Waters Xevo TQ-XS system equipped with a UniSpray™ source was utilized using negative polarity in MRM mode (further details to be found in Supplemental Table S4). All solvents used were UPLC grade and purchased from VWR (Radnor, PA, USA), reference solutions phaseic acid and dihydrophaseic acid were given by the Institute of Experimental Botany, Czech Academy of Science, while abscisic acid was purchased from the Merck-Sigma group (Darmstadt, Germany).

### 3.8. Dry Weight Assessment of Healthy and Botrytized Berry Samples

The dry weight of grape berry samples was determined by keeping 1 g of pulverized grape berry material in a laboratory oven at 105 °C for 1 h and weighing the mass of water loss at the end of the process. Three technical replicates were used for each stage of noble rot.

### 3.9. Determination of Total Antioxidant Capacity of Grape Berries

The total antioxidant capacity of grape berries was determined with an antioxidant assay kit (MAK334, Sigma-Aldrich, St. Louis, MO, USA) according to the manufacturer’s recommendations. During the assay, Cu^2+^ is reduced by antioxidants to Cu^+^, which specifically forms a colored complex with a dye reagent. The color intensity at 570 nm is proportional to the antioxidative capacity of the sample, which is expressed in µM Trolox equivalents. Grape berries were homogenized with liquid nitrogen in mortars, and the tissue suspensions were centrifuged (10,000× *g*, 10 min, 4 °C). Supernatants were diluted with distilled water (1:1), and 80 µL of diluted sample was mixed with 400 µL Reaction Mix. Following 10 min of incubation, the absorbance of solutions was measured at 570 nm. Absorbance values were compared to a calibration curve prepared with 300–1000 µM Trolox solutions.

### 3.10. Data Analysis

Experimental data of ABA, DPA, and total antioxidant quantifications were statistically analyzed by one-way ANOVA and subsequent LSD (Least Significant Difference) test for pairwise comparisons.

## 4. Conclusions

This study gives an insight into two segments of the transcriptome of Furmint berries during natural noble rot development. Exploring results of RNA-Seq analysis, redox- and plant hormone-associated mRNA responses have been highlighted. It has been pointed out that senescence- and stress hormone-connected metabolic pathways are generally activated during noble rot. This is particularly true for ABA, whose early and robust accumulation is probably a crucial molecular cue in the noble rot development of Furmint grape berries. This notion is corroborated by the prompt and uniform upregulation of ABA-metabolic and ABA-responsive transcripts, an extensive gene expression response that does not occur in berries exposed to bunch rot. On the contrary, pathways controlled by juvenile hormones appear either unaffected (cytokinins) or downregulated (auxins) by noble rot. Activation of various *GST* isoforms is also a consistent transcriptional change in berries affected by noble rot. Since noble rot is accompanied by an increase in the level of anthocyanin compounds, GST proteins may participate in transporting anthocyanins from the cytoplasm to the vacuole of plant cells [44,45]. Induction of the phenylpropanoid pathway seems to be a general *B. cinerea* interaction-related plant response (it is also typical of grape berries subjected to bunch rot) with some subtle noble rot-specific features such as the inhibition of *chalcone synthase* isoforms or *Flavonol synthase 1*. It is also accentuated that in some sectors of the grape berry antioxidative apparatus (e.g., two *catalase* isoforms, genes encoding *carotenoid biosynthesis* transcripts), the total antioxidant capacity and transcripts of an array of redox-associated proteins localized in the chloroplasts are consistently suppressed. These noble rot-specific inhibitions might be partly attributed to the apparent color change resulting from the accumulation of quinone and anthocyanin compounds [7,86]. Grape berries are plant organs that conduct photosynthesis [99,100], and extended darkness is known to downregulate transcripts of chloroplastic antioxidants [101].

These findings contribute to the understanding of noble rot, a process vital to the production of botrytized dessert wines in the Tokaj wine region.

## Figures and Tables

**Table 1:** Grapevine genes encoding chloroplast-located redox proteins whose transcription is suppressed in Furmint berries as noble rot progresses.

*A. thaliana* AGI Code	Corresponding *V. vinifera* Ensembl ID	Function	Reference
*At3g50820*	*VIT_18s0001g11710*	Oxygen-evolving enhancer protein 1	[75]
*At3g54050*	*VIT_08s0007g01570*	Fructose-1,6-bisphosphatase	[75]
*At1g32060*	*VIT_02s0109g00080*	Phosphoribulokinase	[75]
*At3g55800*	*VIT_13s0019g03350*	Sedoheptulose-1,7-bisphosphatase	[75]
*At2g39730*	*VIT_06s0004g05180*	Ribulose bisphosphate carboxylase	[75]
*At1g09340*	*VIT_14s0060g00820*	Chloroplast stem-loop binding protein	[75]
*At1g07320*	*VIT_06s0004g06140*	50S ribosomal protein	[76]
*At4g20360*	*VIT_17s0000g09370*	Elongation factor TuB	[76]
*At3g29320*	*VIT_14s0108g01560*	Alpha-1,4 glucan phosphorylase L isozyme	[74]
*At2g39930*	*VIT_11s0078g00310*	Isoamylase 1	[74]
*At1g03310*	*VIT_07s0104g00370*	Isoamylase 2	[74]
*At4g09020*	*VIT_18s0001g06520*	Isoamylase 3	[74]
*At5g64860*	*VIT_07s0031g01540*	4-alpha-glucanotransferase	[74]
*At1g11720*	*VIT_10s0116g01730*	Starch synthase 3	[74]
*At4g18240*	*VIT_11s0065g00150*	Starch synthase 6	[74]
*At3g20440*	*VIT_19s0090g00920*	1,4-alpha-glucan-branching enzyme 1	[74]
*At5g03650*	*VIT_08s0007g03750*	1,4-alpha-glucan-branching enzyme 2	[74]
*At1g44575*	*VIT_18s0001g02740*	Photosystem II 22 kDa protein	[77]
*At1g12900*	*VIT_14s0068g00680*	Glyceraldehyde-3-phosphate dehydrogenase A	[77]
*At1g69830*	*VIT_14s0068g00420*	Alpha-amylase-like 3	[78]
*At5g04140*	*VIT_08s0007g05260*	Ferredoxin-dependent glutamate synthase	[79]
*At1g68830*	*VIT_01s0011g03010*	Serine/threonine-protein kinase STN7	[79]
*At1g20020*	*VIT_04s0023g03510*	Ferredoxin-NADP-oxidoreductase 2	[80]
*At5g38430*	*VIT_17s0000g03690*	Ribulose bisphosphate carboxylase small chain	[81]

## Data Availability

RNA-seq raw data were deposited at ArrayExpress (E-MTAB-11205) and the European Nucleotide Archive (PRJEB48949).

## Supplementary Materials

The following supporting information can be downloaded at: https://www.mdpi.com/article/10.3390/plants11070864/s1, Table S1: Transcript abundance fold change values for genes discussed in this work; Table S2: RNA-Seq validation: primer sequences and Real-time RT-PCR parameters; Table S3: Real-time RT-PCR verification of RNA-Seq results; Table S4: Details of targeted abscisic acid, phaseic acid and dihydrophaseic acid analysis by UPLC-US-MS/MS method.
