# Supplementary material for: Redox and Hormonal Changes in the Transcriptome of Grape (Vitis vinifera) Berries during Natural Noble Rot Development
Source: Plants (Basel). 2022 Mar 24;11(7):864. doi: 10.3390/plants11070864 (PMC9003472; doi:10.3390/plants11070864)
Supplement: Supplementary file 1 [file plants-11-00864-s001.zip › Supplemental File_c_FINAL.pdf]

Supplemental Table S2. RNA-Seq validation: primer sequences and Real-time RT-PCR parameters.

Gene Ensembl ID	Primer sequence	Real-time RT-PCR parameter	
Bcin08g00340	Forward 5' - CGGTTCCCTCAGTTTTGTGT	Polymerase activation and denaturation: 95 °C 2 min	
	Reverse 5' - CGAGACTGAATAACGCGACA		
Bcin13g02250	Forward 5' - TAGGTGACGGCCATTGTGTA		
	Reverse 5' - CGAATACTCCCCGAAACAAA		
Bcin13g02210	Forward 5' - CAGTGCTACCCTTACACCGT	Denaturation: 95 °C 10 sec	Number of cycles used: 40
	Reverse 5' - AGAGTAAGGTTGGGGTCGGT		
Bcin14g03170	Forward 5' - CATTGCCAATATGGATGCTG		
	Reverse 5' - TTGAAGTGCGTTGCTACACC		
Bcin04g00060	Forward 5' - CCTCAGAAGGCACCTCACAA		
	Reverse 5' - GCCCAAGGTTCGCCATTAGA		
VIT_07s0151g00130	Forward 5' - AGCAGCAGTCACAAATTGGC		
	Reverse 5' - GGGCTGAGAAGTTGAGGGAT		
VIT_07s0005g00740	Forward 5' - TCATCACTGCCCTCAATTGC	Annealing and extention: 60 °C 30 sec	
	Reverse 5' - GTCGATGGCTCTGATTGCTC		
VIT_07s0141g00690	Forward 5' - TAGGTTGGATGCGGTGGATT		
	Reverse 5' - TCACCATCAAGCTTCGGAGA		
VIT_06s0004g05700	Forward 5' - GCGAGAGGAAGCGATTGAAG		
	Reverse 5' - AGGCTCCCAACCGAACTTTA		
VIT_05s0062g00310	Forward 5' - TTGGAACCAGAGGCCCTAAG		
	Reverse 5' - TGCTTTGATAACACCGCGAG		

Supplemental Table S3. Real-time RT-PCR verification of RNA-Seq results. Transcript abundance fold-changes between healthy and noble rot-affected Furmint samples are shown after log_2 transformation.

	**Consecutive stages of noble rot**					
	**I.**		**II.**		**III.**	
	**RNA–Seq**	**Real–time RT-PCR**	**RNA–Seq**	**Real–time RT-PCR**	**RNA–Seq**	**Real–time RT-PCR**
VIT_07s0141g00690	5.08	4.80	6.00	6.06	5.31	4.64
VIT_06s0004g05700	4.06	4.13	4.62	4.29	4.85	4.29
VIT_05s0062g00310	6.79	6.88	7.52	7.31	6.75	5.22
VIT_07s0151g00130	3.41	4.74	3.44	4.86	–0.14	0.29
VIT_07s0005g00740	4.88	5.98	5.51	6.76	3.44	3.57
BCIN_08g00340	4.43	3.74	4.85	4.86	1.56	3.11
BCIN_13g02250	4.16	4.64	4.05	4.65	2.74	4.49
BCIN_13g02210	2.84	1.64	3.47	2.19	2.24	1.86
BCIN_14g03170	3.54	3.91	3.27	3.54	2.33	2.85
BCIN_04g00060	0.61	–1.91	1.93	0.17	4.67	3.38
**Correlation**	**0.93**		**0.90**		**0.85**	

Supplemental Table S4. Details of targeted abscisic acid, phaseic acid and dihydrophaseic acid analysis by UPLC-US-MS/MS method - According to Vrhovsek et al 2012. and Pál et al. 2019, with modifications.

**UPLC:**	**Waters Acquity I-class**		
**Column:**	Waters HSS T3 column (1.8 µm, 100 mm × 2.1 mm) at 40°C		
**inj vol.:**	2 µl	**Autosampler temperature:**	10 °C

**Gradient conditions**			
**A: Water (0.1 v/v% formic acid)**		**B: Acetonitrile (0.1 v/v% formic acid)**	
Time (min)	Flow rate (ml/min)	A%	B%
0	0.4	95	5
3	0.4	80	20
4.3	0.4	80	20
9	0.4	55	45
11	0.4	0	100
13	0.4	0	100
13.01	0.4	95	5
15	0.4	95	5

**Detector:**	**Xevo TQ-XS with Unispray source**
**Resolution:**	Unit mass (+/- 0.8 Da)
**Impactor voltage:**	2 kV
**Desolvation temperature:**	550°C
**Nebulizer gas:**	6.5 bar N_2
**Desolvation gas flow:**	1000 L/h N_2
**Cone gas flow:**	450 L/h N_2
Collision gas flow:	0.15 ml/min Argon 5.0

**Component name**	**Unispray Impactor voltage polarity**	**quant. MS/MS transition**	**Pred.RT (min)**	**RT found (min)**	**Cone voltage (V)**	**Collision ennergy (eV)**	**Calibrated to**	**QC**
**[2H6](+)-cis,trans-abscisic acid Internal standard**	negative	269.1 > 159.1	7.13	7.13	20	10	external	spike recovery
**abscisic acid**	negative	**263.1 > 153**	**7.18**	**7.16**	20	10	external	IS recovery and spike recovery
**phaseic acid**	negative	**279.12 > 139**	**5.24**	**5.18**	25	12	external	spike recovery
**dihydrophaseic acid**	negative	**281.14 > 237.2**	**3.56**	**3.52**	25	12	external	spike recovery

References:

Vrhovsek, U., Masuero, D., Gasperotti, M., Franceschi, P., Caputi, L., Viola, R., et al. (2012). A versatile targeted metabolomics method for the rapid quantification of multiple classes of phenolics in fruits and beverages. J. Agric. Food Chem. 60, 8831–8840. doi:10.1021/jf2051569.

Pál, M., Ivanovska, B., Oláh, T., Tajti, J., Hamow, K. Á., Szalai, G., et al. (2019). Role of polyamines in plant growth regulation of *Rht* wheat mutants. Plant Physiol. Biochem. 137, 189–202. doi:10.1016/j.plaphy.2019.02.013.
